# Expressional Profiling of Carpet Glia in the Developing *Drosophila* Eye Reveals Its Molecular Signature of Morphology Regulators

**DOI:** 10.3389/fnins.2019.00244

**Published:** 2019-03-29

**Authors:** Tsung-Ying Ho, Wei-Hang Wu, Sheng-Jou Hung, Tsunglin Liu, Yuan-Ming Lee, Ya-Hsin Liu

**Affiliations:** ^1^Department of Life Sciences, College of Bioscience and Biotechnology, National Cheng Kung University, Tainan, Taiwan; ^2^Department of Biotechnology and Bioindustry Sciences, College of Bioscience and Biotechnology, National Cheng Kung University, Tainan, Taiwan; ^3^Institute of Molecular Biology, Academia Sinica, Taipei, Taiwan

**Keywords:** carpet glia, subperineurial glia, blood–brain barrier, targeted DamID-seq, transcriptome

## Abstract

Homeostasis in the nervous system requires intricate regulation and is largely accomplished by the blood–brain barrier (BBB). The major gate keeper of the vertebrate BBB is vascular endothelial cells, which form tight junctions (TJs). To gain insight into the development of the BBB, we studied the carpet glia, a subperineurial glial cell type with vertebrate TJ-equivalent septate junctions, in the developing *Drosophila* eye. The large and flat, sheet-like carpet glia, which extends along the developing eye following neuronal differentiation, serves as an easily accessible experimental system to understand the cell types that exhibit barrier function. We profiled transcribed genes in the carpet glia using targeted DNA adenine methyl-transferase identification, followed by next-generation sequencing (targeted DamID-seq) and found that the majority of genes expressed in the carpet glia function in cellular activities were related to its dynamic morphological changes in the developing eye. To unravel the morphology regulators, we silenced genes selected from the carpet glia transcriptome using RNA interference. The *Rho1* gene encoding a GTPase was previously reported as a key regulator of the actin cytoskeleton. The expression of the *pathetic* (*path*) gene, encoding a solute carrier transporter in the developing eye, is specific to the carpet glia. The reduced expression of *Rho1* severely disrupted the formation of intact carpet glia, and the silencing *path* impaired the connection between the two carpet glial cells, indicating the pan-cellular and local effects of Rho1 and Path on carpet glial cell morphology, respectively. Our study molecularly characterized a particular subperineurial cell type providing a resource for a further understanding of the cell types comprising the BBB.

## Introduction

The blood–brain barrier (BBB), the cellular interface between the brain and blood, is essential for the normal functionality of the nervous system. The BBB forms a selective shield between the nervous and circulatory systems and controls the passage of molecules, including ions, nutrients, and neurotoxins, as well as the trafficking of lymphocytes for immune surveillance and responses (Abbott et al., 2010; Obermeier et al., 2013). The role of the BBB in response to environmental stimuli is not only limited to its barrier function. It has been shown that the *Drosophila* BBB responds to nutritional signals and produces insulin-like peptides that trigger the reactivation of neural stem cells from a quiescent state (Spéder and Brand, 2014). Moreover, BBB breakdown has been associated with several neurodegenerative diseases (Obermeier et al., 2013; Hagan and Ben-Zvi, 2015). BBB glia is most susceptible to a poly-glutamine (polyQ)-induced neurodegenerative disease model in *Drosophila* (Yeh et al., 2018).

The vertebrate and *Drosophila* BBBs share many structural and functional characteristics (Banerjee and Bhat, 2007; DeSalvo et al., 2011, 2014; Hindle and Bainton, 2014). The vertebrate BBB is primarily composed of a specialized capillary endothelium in the brain vasculature. These endothelial cells form tight junctions (TJs) along their length that enable the barrier function of the BBB and are surrounded by pericytes secreting extracellular matrix and astrocytes delivering nutrition from the blood to the neurons (Freeman and Doherty, 2006; Abbott et al., 2010). The *Drosophila* BBB is composed of layers of flattened glial cells that separate the brain from the hemolymph (Stork et al., 2008). The outermost glial cell layer, the perineurial glia (PG) and their surrounding extracellular matrix, called neural lamella, mediates barrier selectivity, while the underlying subperineurial glial cells (SPGs), functionally and structurally equivalent to the capillary endothelium of the vertebrate BBB, form septate junctions (SJs) that serve as an insulator function (Stork et al., 2008; Limmer et al., 2014).

In the developing *Drosophila* eye, a particular cell type, the carpet glia, is defined as SPG based on its expression of the *moody* gene, which encodes G-protein coupled receptors (GPCRs) required for SJ formation (Bainton et al., 2005; Schwabe et al., 2005; Silies et al., 2007). In addition to the expression of *moody* and formation of SJs, the large and flattened morphology of carpet glia also conforms to that of the SPGs in the *Drosophila* brain BBB (Stork et al., 2008). It has been shown that two carpet cells are present in the optic stalk in second instar larvae with retinal basal glia (RBG), and they migrate into the eye imaginal disc at the early third instar stage (Choi and Benzer, 1994; Silies et al., 2007). Upon migration into the eye imaginal disc, the two large nuclei of carpet glia remain bilateral in the posterior margin of the eye disc, while their membrane extends anteriorly, such that each carpet glial cell covers one half of the differentiated eye field (Silies et al., 2007). The RBGs migrate into the eye disc in response to the differentiating photoreceptor cells (PRs), and their contact with PR axons induces them to differentiate into wrapping glial cells (WG), which ensheath PR axons and guide the axons into the brain (Rangarajan et al., 1999; Hummel et al., 2002). The migratory RBGs follow the differentiating PRs and are usually two to three cells posterior to the front of the PRs. When carpet glial cells were ablated by the cell-type-specific expression of apoptosis genes, RBGs migrated further and became positioned anteriorly to the front of the differentiating PRs, suggesting that carpet glia function in restraining RBG migration (Silies et al., 2007).

The unique morphology of carpet glia and the clear functional readout of overmigratory RBGs in its absence, make the carpet glia a proper model system to study the SPG type, which constitutes the primary component of the *Drosophila* BBB. In this study, we use “TaDa,” targeted DNA adenine methyl-transferase identification followed by next-generation sequencing (targeted DamID-seq; Southall et al., 2013), to profile the gene expression of carpet glia. The identification of numerous characteristic genes of the BBB function validates the use of carpet glia as an experimental system for studying the subperineurial cell type. The gene ontology (GO) term analyses of the carpet glia transcriptome revealed that the majority of genes expressed in the carpet glia function in cellular activities that require dynamic morphological changes and involve the continuous reorganization of the cytoskeleton. By specifically knocking down gene expression in the carpet glia, we show that two genes, *Rho1*, which encodes a GTPase functioning as a major regulator of the actin cytoskeleton, and the carpet glia-specific *pathetic* (*path*) gene encoding a transmembrane amino acid transporter, are required for the formation of intact carpet glia. Using dye permeability assays, we demonstrate that the BBB integrity is disrupted upon *Rho1* knockdown in the carpet glia, suggesting that the development of carpet glia could affect BBB function. Altogether, we present a validated carpet glia transcriptome that facilitates future studies on the development of the BBB.

## Materials and Methods

### Targeted DamID-seq and Analysis

*UAS-LT3-NDam* and *UAS-LT3-NDam-RpII215* (Southall et al., 2013) were crossed with *C135-Gal4* (Hrdlicka et al., 2002). Embryos were collected over a 4-h period at 25°C and then shifted to 29°C for 4 days. A total of 5,000 eye discs from mid- to late-third instar larvae were dissected for each sample and two biological replicates were performed for each genotype. Genomic DNA was extracted from the dissected third instar larval eye discs and methylated DNA was processed and amplified as described by Marshall et al. (2016). Sequencing libraries of the amplified DNA and subsequent Illumina HiSeq 2000 paired-end sequencing were prepared and performed, respectively, according to the manufacturer's protocols by the National Center for Genome Medicine, Taipei, Taiwan.

The genomic regions bound by Dam-Pol II or Dam proteins were identified using damidseq_pipeline v1.4 (Marshall and Brand, 2015), which required bowtie2 and SAMtools (Li et al., 2009; Langmead and Salzberg, 2012). In addition, we downloaded prebuilt GATC fragment files (*D. melanogaster* genome BDGP R.6/dm6) provided by the pipeline. We used bowtie2 v2.2.5 to align DamID sequencing paired-end reads to the *Drosophila* genome BDGP R.6/dm6. The bowtie2 options -I/–minins and -X/–maxins were set as the minimum/maximum fragment lengths of the DamID sequencing data as sample_1_Dam_biological replicate 1 = 178/1,082, sample_2_Dam-Pol II_biological replicate 1 = 162/1,282, sample_3_Dam_biological replicate 2 = 124/1,005, and sample_4_Dam-Pol II_biological replicate 2 = 164/1,300. The alignment results were converted from the SAM format into BAM files using SAMtools v1.1. With the BAM files, the damidseq_pipeline v1.4 with default options outputted bedgraph files, which represented the Dam-Pol II/Dam ratios on a log_2_ scale at all GATC fragments. We used the R script polii.gene.call and a GFF file containing the gene annotation and position information of the *Drosophila* genome BDGP R.6/dm6, both provided by the damidseq_pipeline, to assign Dam-Pol II binding regions to genes. The output genes.details files showed the average Pol II occupancy and false discovery rate (FDR) value. In addition, we converted bedgraph files to the tdf format by using a gff2tdf perl script provided in the damidseq_pipeline and visualized the Dam-Pol II binding in the Integrative Genomics Viewer (IGV) tool v2.4.15 (Thorvaldsdóttir et al., 2013).

### Fly Stocks

All flies were raised with standard procedures. *UAS-LT3-NDam* and *UAS-LT3-NDam-RpII215* (Southall et al., 2013) were provided by Dr. Andrea Brand. *C135-Gal4* (DGRC #108995; Hrdlicka et al., 2002) was obtained from the Kyoto Stock Center (DGRC). *UAS-mCD8GFP* (BDSC #5137), *UAS-DsRed* (BDSC #6280), and the protein-tag line of the *kay* gene, Mi{PT-GFSTF.0}kay[MI05333-GFSTF.0] (BDSC #63175), were obtained from Bloomington *Drosophila* Stock Center (BDSC). For the knockdown experiments using *C135-Gal4* driving dsRNA constructs, larvae were incubated at 25°C before the second instar and then shifted to 29°C until dissection at the mid-third instar stage. The different *UAS-dsRNA* flies used in this study were obtained from the Vienna *Drosophila* Resource Center (VDRC) or the TRiP collection (BDSC): *UAS-kay* ^*dsRNA*^ (BDSC #27722, #31391), *UAS-Rho1* ^*dsRNA*^ (BDSC #9909, #9910), and *UAS-path* ^*dsRNA*^ (VDRC #100519). Other fly lines used were *UAS-Upd3* (Houtz et al., 2017; provided by Dr. Yu-Chen Tsai), *10xSTAT-GFP* (Bach et al., 2007; provided by Dr. Y. Henry Sun), and *10xSTAT-GFP-nls* (Tsai et al., 2015; provided by Dr. Yu-Chen Tsai).

### Genomic Mapping of the *C135-Gal4* Fly Line

The *C135-Gal4* fly line contains a transposon P element, P{GawB}. Genomic DNA of the *C135-Gal4* flies was extracted and digested with the restriction enzyme NcoI at 37°C overnight. The digested DNA fragments were cloned and sequenced using the primer 5′-CAATCATATCG CTGTCTCACTCA-3′. Sequencing results were blasted to identify the P element insertion site.

### Gene Ontology Term Analysis

GO annotation was performed using default settings in the DAVID Bioinformatics web server (https://david.ncifcrf.gov, Database for Annotation, Visualization and Integrated Discovery v6.8; Huang et al., 2009a,b). Gene lists submitted to DAVID for GO term analysis are shown in Tables S1–S3.

### Immunostaining and Imaging

Eye discs were dissected out in phosphate-buffered saline (PBS) and then fixed in 4% paraformaldehyde for 15 min. After permeabilization with PBS/0.1% Triton X-100 for 1 h, samples were blocked in buffer [5% bovine serum albumin (BSA), 5% goat serum/PBS/0.1% Tween 20] for 1 h and then incubated with primary antibody diluted in blocking buffer overnight at 4°C. After three washes in PBS, the samples were incubated with the appropriate secondary fluorescent antibody (Jackson) for 2 h at room temperature. The primary antibodies were mouse anti-Repo (DHSB), rat anti-Elav (DHSB), chicken anti-GFP (Abcam), rabbit anti-DsRed (Clontech), and goat anti-HRP (Cappel). Images were obtained with a Zeiss LSM 780 confocal microscope.

### Blood–Retina Barrier Permeability Assay

The permeability assay was as previously described (Yeh et al., 2018). *C135-Gal4*; *UAS-mCD8GFP* females were crossed with *UAS-Rho1*^*dsRNA*^ males at 25°C, and the progeny embryos were raised at 29°C. *C135-Gal4*; *UAS-mCD8GFP* flies were used as controls and raised in parallel under the same conditions. Two-day-old adults were used for the permeability assay. FlyNap (triethylamine)-anesthetized adult flies were injected with thin borosilicate needles containing 50 mg/ml tetramethyl-rhodamine dextran (MW 10000, Molecular Probes, #D1816) under a dissecting microscope. Approximately 10 nl of the dye was injected into the soft tissue between the exoskeleton of two abdominal segments of the adult flies. After a 2-h recovery, the eyes of live adult flies were examined and photographed with a Zeiss LSM 880 confocal microscope. Quantification of dye leakage in each eye was measured by the average fluorescence intensity over the whole eye and normalized against the fluorescence intensity of the antenna using Image J.

## Results

### Targeted DamID-Sequencing to Profile Carpet Glia Expression

**Figure 1:**
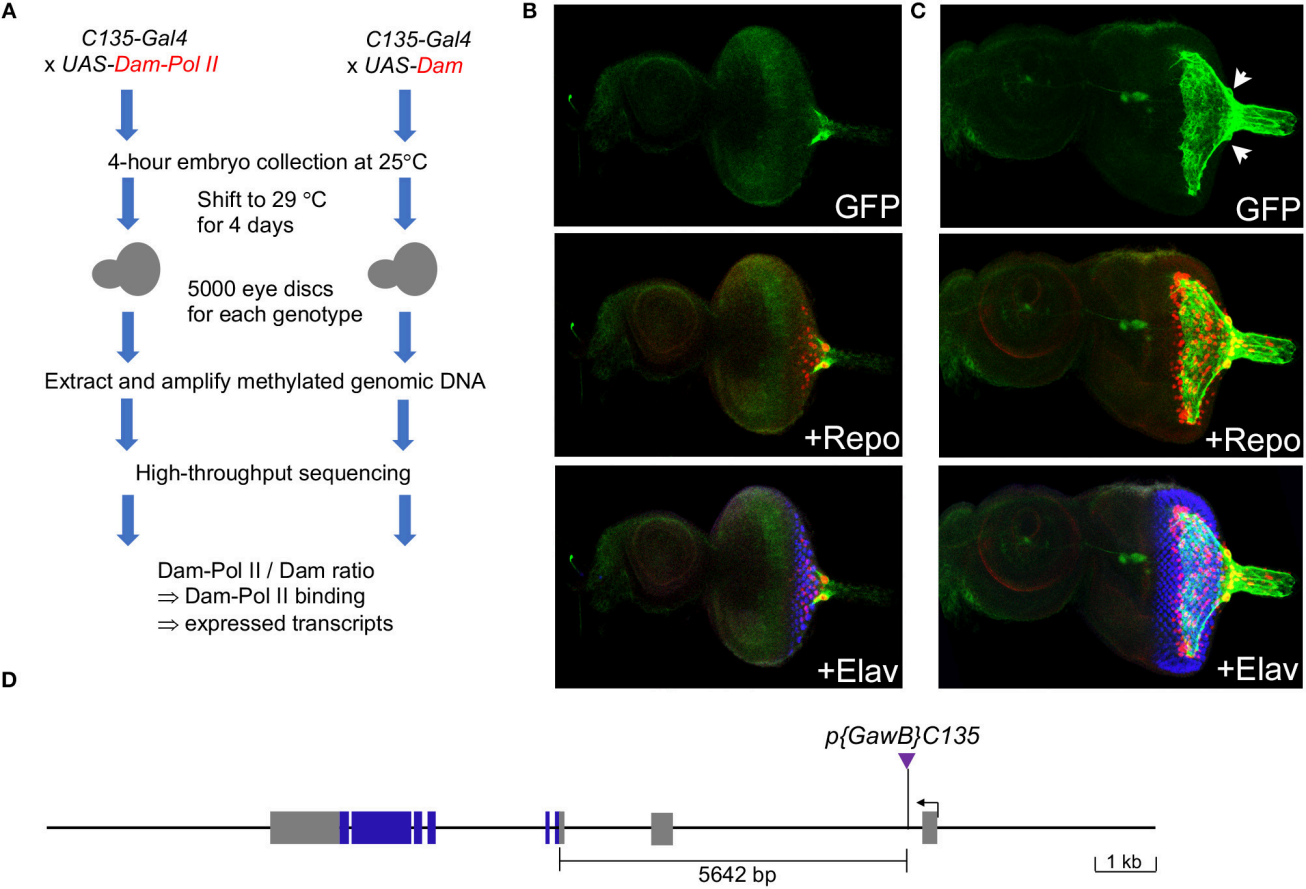
Characterization of the *C135-Gal4* driver used in this study. **(A)** Experimental design: the *C135-Gal4* driver was used to drive the expression of Dam-Pol II and Dam. There was no temporal control of Gal4 expression as *C135-Gal4* drives no expression in the embryonic eye field. **(B,C)** Expression of the *C135-Gal4* driver in the eye discs of early-third **(B)** and mid-third **(C)** instar larvae. All images are z-projection of confocal sections. Larval eye imaginal disc stained for the presence of carpet glia membrane (GFP staining, green), glial nuclei (Repo staining, red), and differentiated PRs (Elav staining, blue). **(D)** Insertion of *C135-Gal4* in the *path* locus. The organization of the *path* locus is depicted. *C135-Gal4*, indicated by a purple triangle, is inserted 5642 bp upstream of the translational start site.

To identify genes expressed in the carpet glia, we used targeted DamID-seq (Southall et al., 2013). This method utilizes the Gal4-UAS system and can profile the genome-wide binding of RNA polymerase II (Pol II), which is tagged by adenine methylation in a cell-type-specific manner. We used the *C135-Gal4* driver, which is specifically expressed in the carpet glia (Tsao et al., 2016; Yeh et al., 2018), to drive the expression of *UAS-Dam-Pol II* and *UAS-Dam-only* (Figure 1A). Immunostaining of eye discs carrying *C135-Gal4* driving a membrane-bound GFP reporter gene, *UAS-mCD8GFP*, showed that *C135-Gal4* is expressed in cells with an extensive mesh-like plasma membrane covering the third instar larval eye disc, and two large nuclei were clearly outlined (Figure 1C, compared to Figure 1B, the early third instar), revealing the unique cellular morphology of carpet glia (Silies et al., 2007; Yuva-Aydemir et al., 2011). We mapped the *C135-P[Gal4]* insertion to the first intron of the *pathetic* (*path*) gene (Figure 1D).

We drove the expression of Dam-Pol II and Dam-only with *C135-Gal4* from embryos to mid-third instar larvae. The potential toxicity of the high-level of DNA adenine methyltransferase (van Steensel and Henikoff, 2000) during the constant expression from embryos to larvae was examined by immunostaining of eye discs carrying *C135Gal4* driving *UAS-mCD8GFP* and *UAS-Dam-Pol II* (or *UAS*-*Dam-only*) together. We found that the extensive membrane morphology of carpet glia was intact, indicating that the differentiation of carpet glia was not compromised. The embryonic expression of *C135-Gal4* in a subset of proventriculus (Hrdlicka et al., 2002) should not affect the transcriptional profile of carpet glia. The RNA Pol II binding profile obtained in the *C135-Gal4*-driven DamID-seq experiments hence represents the genes transcribed during the entire developmental process of carpet cells from their birth in the optic stalk to their entry into the eye disc with extending membrane.

To examine the Dam-Pol II occupancy in the carpet glia, we utilized the bioinformatics tool “damidseq_pipeline” developed by the Brand lab accompanying the TaDa method (Marshall and Brand, 2015), which determines the mean ratios of Dam-Pol II to Dam-only binding across annotated transcripts and assigns an FDR to each transcript. Comparison of the intensity of each Dam-Pol II to Dam-only binding peak at the same genomic location between two biological replicates revealed a high degree of reproducibility, with a correlation coefficient of 0.92 (Figure S1A). We considered transcripts with an FDR < 1% in both biological replicates of Dam-Pol II to Dam-only and an average log_2_ ratio change of the two biological replicates >0.5 as significantly bound by Dam-Pol II and therefore “expressed” in the carpet glia. Using these criteria, we found that the *path* gene, where the *C135-Gal4* insertion resides and known to be expressed in the carpet glia, was included in the expressed gene list of carpet glia, validating our results. In total, we identified 3,469 genes with significant Pol II occupancy (Table S1). Several genes, including *moody, Neurexin IV* (*Nrx-IV*), *spinster* (*spin*), *cut, apontic* (*apt*), *Gliotactin* (*Gli*), and ö*bek*, have been reported to be expressed in the subperineurial glia (Sepp and Auld, 1999; Bainton et al., 2005; Schwabe et al., 2005; Stork et al., 2008; Yuva-Aydemir et al., 2011; Bauke et al., 2015; Sasse and Klämbt, 2016; Zülbahar et al., 2018). We found Pol II binding to the genes *moody* and *spin*, which are known to be expressed in the carpet glia (Figures S1B,C), and also *apt, Gli*, and ö*bek*, which are known to be expressed in other SPGs. The high coverage of subperineurial glia-expressing genes validates the Pol II binding profile of carpet glia.

### Carpet Glia Transcriptome

**Figure 2:**
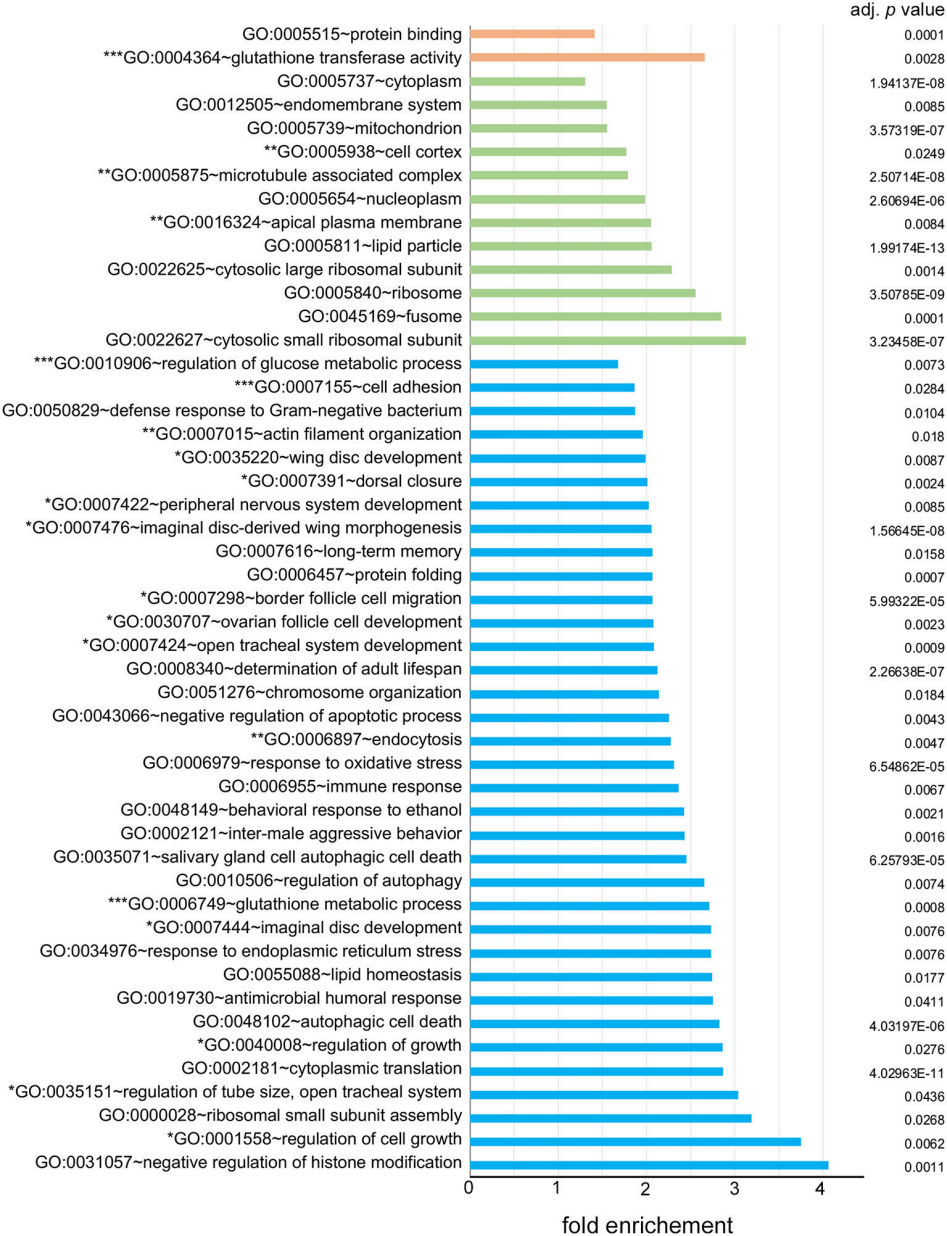
Over-represented gene ontology (GO) terms of the carpet glia transcriptome. All GO terms in three fields, biological process (BP) labeled in blue, cellular component (CC) labeled in green, and molecular function (MF) labeled in orange, with a Benjamini-adjusted *p* < 0.05 are listed. Enrichment shown on the *x*-axis represents the fold increase in the number of genes in each GO term over the number expected by chance. GO terms with one, two, and three asterisks (^*^) are categorized in groups 1, 2, and 3 described in the text, respectively.

To elucidate the characteristics of genes expressed in the carpet glia, we focused on the 2,689 protein-coding genes and used DAVID bioinformatics tools to functionally annotate each gene for its GO terms in aspects of the biological process (BP), molecular function (MF), and cellular component (CC; Table S2; Huang et al., 2009a,b). Moreover, we examined the enrichment of specific GO terms of these genes. Figure 2 lists all over-represented GO terms in all three categories with a Benjamini-adjusted *p* < 0.05.

We categorized the over-represented GO terms into three groups: (1) GO terms associated with growth, imaginal disc development, and cell movement. GO terms such as wing disc development, imaginal disc-derived wing morphogenesis, border cell migration, and dorsal closure were included in this group. (2) GO terms associated with molecules in the cytoskeleton, such as actin filament organization and microtubule associated complex. The cellular activities suggested by the over-represented GO terms in the first two groups are consistent with those of carpet cells, whose large and flat cell bodies extend anteriorly in the developing eye and hence undergo continuous morphological changes. (3) GO terms associated with the BBB functions, including cell adhesion, regulation of glucose metabolism, glutathione transferase activity, and the glutathione metabolic process. The over-represented GO terms of these groups suggest a potential function of carpet glia as a chemical protection interface.

### Comparison With *Drosophila* and Mouse BBB Transcriptomes

DeSalvo et al. (2014) purified the adult *Drosophila* BBB surface glia, based on expression of *9-137-Gal4* in both PG and SPG, with fluorescence activated cell sorting (FACS) and profiled the gene expression with microarrays. To affirm that carpet glia is a valid model system for studying the subperineurial cell type, we compared the Dam-Pol II binding data of carpet glia to the enriched genes in the adult BBB transcriptome (DeSalvo et al., 2014). We compiled a non-redundant gene list containing 1,273 genes by combining the genes enriched in surface glia relative to all brain glia, neurons, and whole brains from the surface glia microarray datasets (DeSalvo et al., 2014). Comparison between the transcriptome of adult surface glia and the 2,689 protein-coding genes bound by Dam-Pol II in carpet glia yielded 456 common genes (Table S3), which represents a significant portion shared by adult surface glia and carpet glia (Fisher's exact test, *p* < 2.2E−16). In addition to the functional annotation of these genes in three GO term fields, we also used DAVID bioinformatics tools to examine the biological pathways (KEGG Pathway Database) and protein domains (Interpro) of the 456 common genes. We examined whether characteristic genes for BBB functions are present among the 456 common genes. The characteristic genes for BBB functions include those (1) involved in the formation of SJs that perform the barrier function, (2) encoding enzymes catalyzing the metabolism of toxic compounds, and (3) encoding ATP-binding cassette (ABC) transporters that export toxic metabolites out of the brain (Abbott et al., 2010; DeSalvo et al., 2014). All three categories of characteristic BBB genes were expressed in the carpet glia as in the adult BBB (Table 1). For example, Moody and Locomotion defects (Loco), components of GPCR signaling required for SJ formation, were identified (Schwabe et al., 2005). A large number of oxidation–reduction enzymes such as cytochrome P450 and glutathione transferases that catalyze toxic compounds and ABC transporters for toxin efflux were also found. Our Dam-Pol II-bound transcriptome therefore provides a molecular basis supporting the notion that the carpet cells are among the subperineurial cells, which are the primary component of the *Drosophila* BBB.

**Table 1:** Common genes in the transcriptomes of carpet glia, adult surface glia, and the mouse BBB.

**Category**	**Term**	**Gene name (symbol)**	**Average log_**2**_ FC**	**Molecular function**	**Mouse BBB gene Gene name (symbol)**
**CELL ADHESION MOLECULES**					
GOTERM_MF_DIRECT	GO:0050839~cell adhesion molecule binding	*midline fasciclin* (*mfas*)	1.26	GO:0050839~cell adhesion molecule binding	
GOTERM_BP_DIRECT	GO:0007155~cell adhesion	*Rho-Like* (*RhoL*)	1.07	GO:0003924~GTPase activity	
		*held out wings* (*how*)	0.72	GO:0003730~mRNA 3′-UTR binding	
		CAP	0.72	InterPro: IPR028516~actin filament organization	Sorbin and SH3 domain containing 2 (Sorbs2)
		*scab* (*scb*)	0.70	GO:0050839~cell adhesion molecule binding	
		*pebble* (*pbl*)	0.60	GO:0005096~GTPase activator activity	
		*Notch* (*N*)	0.54	GO:0004888~transmembrane signaling receptor activity	
GOTERM_BP_DIRECT	GO:0019991~septate junction assembly	*lethal (2) giant larvae* (*l(2)gl*)	0.83	GO:0005096~GTPase activator activity	LLGL1 scribble cell polarity complex component (Llgl1)
		*locomotion* defects (*loco*)	0.79	GO:0001965~G-protein alpha-subunit binding	Regulator of G-protein signaling 5 (Rgs5)
		*moody*	0.70	GO:0004930~G-protein coupled receptor activity	
		*Neuroglian* (*Nrg*)	0.60	GO:0005509~calcium ion binding	F11 receptor (F11r)
		*coracle* (*cora*)	0.55	GO:0005200~structural constituent of cytoskeleton	
GOTERM_CC_DIRECT	GO:0005912~adherens junction	*Moesin* (*Moe*)	1.43	GO:0003779~actin binding	
		*Shroom*	0.84	GO:0003779~actin binding	Shroom family member 4 (Shroom4)
		*shotgun* (*shg*)	0.76	GO:0008013~beta-catenin binding	
		*warts* (*wts*)	0.75	GO:0004674~protein serine/threonine kinase activity	
		*karst* (*kst*)	0.59	GO:0008092~cytoskeletal protein binding	
GOTERM_CC_DIRECT	GO:0005925~focal adhesion	*blistery* (*by*)	1.25	GO:0003779~actin binding	
		*inflated* (*if*)	0.74	GO:0050840~extracellular matrix binding	
		*Vinculin* (*Vinc*)	0.65	GO:0003779~actin binding	Catenin, cadherin associated protein, alpha 1 (Ctnna1)
		*alpha actinin* (*Actn*)	0.60	GO:0003779~actin binding	
		*Paxillin* (*Pax*)	0.60	GO:0008270~zinc ion binding	
**TRANSPORTERS**					
GOTERM_BP_DIRECT	GO:0006810~transport	*Acyl-CoA binding protein 2* (*Acbp2*)	1.39	GO:0000062~fatty-acyl-CoA binding	Diazepam binding inhibitor (Dbi)
		*CG6836*	1.17	GO:0005215~transporter activity	
		*CG3091*	1.04	GO:0005215~transporter activity	
		*bloated tubules* (*blot*)	0.95	GO:0005215~transporter activity	
		*CG3792*	0.90	UniProtKB:O75352~protein glycosylation	
		*CG12004*	0.69	GO:0005215~transporter activity	
		*inebriated* (*ine*)	0.60	GO:0005215~transporter activity	
		*bedraggled* (*bdg*)	0.56	GO:0005215~transporter activity	
INTERPRO	IPR013525:ABC-2 type transporter	*CG3164*	1.13	GO:0016887~ATPase activity	ATP binding cassette subfamily G member 2 (Abcg2)
		*CG9990*	1.01	GO:0016887~ATPase activity	ATP binding cassette subfamily G member 2 (Abcg2)
		*CG31689*	0.76	GO:0016887~ATPase activity	ATP binding cassette subfamily G member 2 (Abcg2)
INTERPRO	IPR020846:Major facilitator superfamily domain	*Picot*	1.39	GO:0015293~symporter activity	
		*Organic anion transporting polypeptide 74D* (*Oatp74D*)	0.91	GO:0005215~transporter activity	Solute carrier organic anion transporter family, member 2b1 (Slco2b1)
		*CG8034*	0.89	GO:0008028~monocarboxylic acid transmembrane transporter activity	Solute carrier family 16, monocarboxylic acid transporter, member 4 (Slc16a4)
		*CG15890*	0.78	InterPro: IPR011701~transmembrane transport	Solute carrier family 16, member 3 (Slc46a3)
		*CG6231*	0.72	GO:0015101~organic cation transmembrane transporter activity	Solute carrier family 22, organic anion transporter, member 8 (Slc22a8)
		*CG3168*	0.71	GO:0008514~organic anion transmembrane transporter activity	Solute carrier family 2, facilitated glucose transporter, member 1 (Slc2a1)
		*Organic cation transporter 2* (*Orct2*)	0.71	GO:0022857~transmembrane transporter activity	Solute carrier family 22, organic anion transporter, member 8 (Slc22a8)
		*CG4797*	0.58	GO:0022857~transmembrane transporter activity	
		*CG3036*	0.58	GO:0022857~transmembrane transporter activity	
		*CG6126*	0.52	GO:0005275~amine transmembrane transporter activity	Solute carrier family 22, organic anion transporter, member 8 (Slc22a8)
		*CG8051*	0.50	GO:0008028~monocarboxylic acid transmembrane transporter activity	Solute carrier family 16, monocarboxylic acid transporter, member 1 (Slc16a1)
**DRUG METABOLISM**					
KEGG_PATHWAY	dme00040:Pentose and glucuronate interconversions	*Glutathione S transferase S1* (*GstS1*)	1.66	GO:0004364~glutathione transferase activity	Glutathione S-transferase, mu 2 (Gstm2)
GOTERM_BP_DIRECT	GO:0006098~pentose-phosphate shunt	*Chloride intracellular channel* (*Clic*)	1.55	GO:0005254~chloride channel activity	
KEGG_PATHWAY	dme00480:Glutathione metabolism	*Glutathione S transferase D9* (*GstD9*)	1.52	GO:0004364~glutathione transferase activity	
GOTERM_MF_DIRECT	GO:0004602~glutathione peroxidase activity	*Glutathione S transferase D1* (*GstD1*)	1.44	GO:0004364~glutathione transferase activity	
GOTERM_MF_DIRECT	GO:0004364~glutathione transferase activity	*CG9436*	1.42	GO:0004032~alditol:NADP+1-oxidoreductase activity	Aldo-keto reductase family, member C14 (Akr1c14)
GOTERM_BP_DIRECT	GO:0006749~glutathione metabolic process	*CG3609*	1.39	GO:0016491~oxidoreductase activity	
INTERPRO	IPR010987:Glutathione S-transferase, C-terminal-like	*CG10863*	1.29	GO:0004032~alditol:NADP+1-oxidoreductase activity	Aldo-keto reductase family, member C14 (Akr1c14)
INTERPRO	IPR004046:Glutathione S-transferase, C-terminal	*Glutathione S transferase E3* (*GstE3*)	1.28	GO:0004364~glutathione transferase activity	
INTERPRO	IPR004045:Glutathione S-transferase, N-terminal	*Phosphogluconate dehydrogenase* (*Pgd*)	1.19	GO:0004616~phosphogluconate dehydrogenase activity	
KEGG_PATHWAY	dme00982:Drug metabolism-cytochrome P450	*Thioredoxin peroxidase 1* (*Jafrac1*)	1.15	GO:0004602~glutathione peroxidase activity	
KEGG_PATHWAY	dme00980:Metabolism of xenobiotics by cytochrome P450	*Glutathione S transferase E6* (*GstE6*)	1.17	GO:0004364~glutathione transferase activity	
		*Microsomal glutathione S-transferase-like* (*Mgstl*)	1.17	GO:0004364~glutathione transferase activity	
		*CG30022*	0.97	GO:0005506~iron ion binding	
		*CG30499*	0.88	GO:0004750~ribulose-phosphate 3-epimerase activity	
		*Glutamate-cysteine ligase catalytic subunit* (*Gclc*)	0.71	GO:0004357~glutamate-cysteine ligase activity	
		*CG17323*	0.59	GO:0015020~glucuronosyltransferase activity	
		*CG6084*	0.59	GO:0004032~alditol:NADP+1-oxidoreductase activity	Aldo-keto reductase family, member C14 (Akr1c14)
		*UDP-glycosyltransferase family 35 member A1* (*Ugt35A1*)	0.54	GO:0008194~UDP-glycosyltransferase activity	

*All genes annotated in distinct GO term categories, KEGG pathways, and Interpro are listed. Genes in the fields of cell adhesion molecules and transporters are further divided based on their annotated GO terms. Genes in the field of drug metabolism are listed without further division to avoid redundancy. For simplicity, only one GO_MF term for each gene is displayed. For details, please see Table S3. BBB, blood–brain barrier*.

Daneman et al. (2010) utilized FACS and antibody depletion to purify endothelial cells from a mouse brain, liver, and lung and profiled the gene expression of each cell population with microarrays. They obtained genes enriched in the BBB-forming endothelial cells of the mouse brain by comparing the transcriptional profile of brain endothelial cells with those of liver and lung endothelial cells. To determine which among the genes common to both carpet glia and adult surface glia are conserved between species, we compared the 456 common genes with the mouse BBB transcriptome (Daneman et al., 2010). We searched mouse orthologs of the 456 *Drosophila* genes using DIPOT (DRSC integrative ortholog prediction tool; Hu et al., 2011) and also the fly-to-mouse protein sequence alignment results presented in DeSalvo et al. (2014). Comparison of the mouse orthologs of these *Drosophila* genes with mouse BBB-enriched genes showed that the predicted mouse orthologs of 59 *Drosophila* genes are present in the mouse BBB transcriptome (Table S3). Several of the conserved genes encode characteristic BBB proteins, including SJ/TJ complexes, transporters, and metabolic enzymes (Table 1). Both Loco, a regulator of G-protein signaling, and Moody encoding G protein-coupled receptors are essential for *Drosophila* SJ formation and are bound by Dam-Pol II in the carpet glia. We found that only the predicted mouse ortholog of Loco, Rgs 5, but not that of Moody, is present in the mouse BBB transcriptome. Moreover, the *Drosophila* gene *CG3168* identified in the carpet glia transcriptome is closely related to the mouse solute carrier SLC2A1 (i.e., GLUT-1), which is a well-characterized transporter shuttling glucose between the blood and the brain (Boado and Pardridge, 1990; Pardridge et al., 1990). The presence of these conserved genes in both the carpet glia and mouse BBB shows that carpet glial cells share some molecular features with mammalian BBB endothelial cells.

### Genes Function in Regulating Carpet Glial Cell Morphology

**Figure 3:**
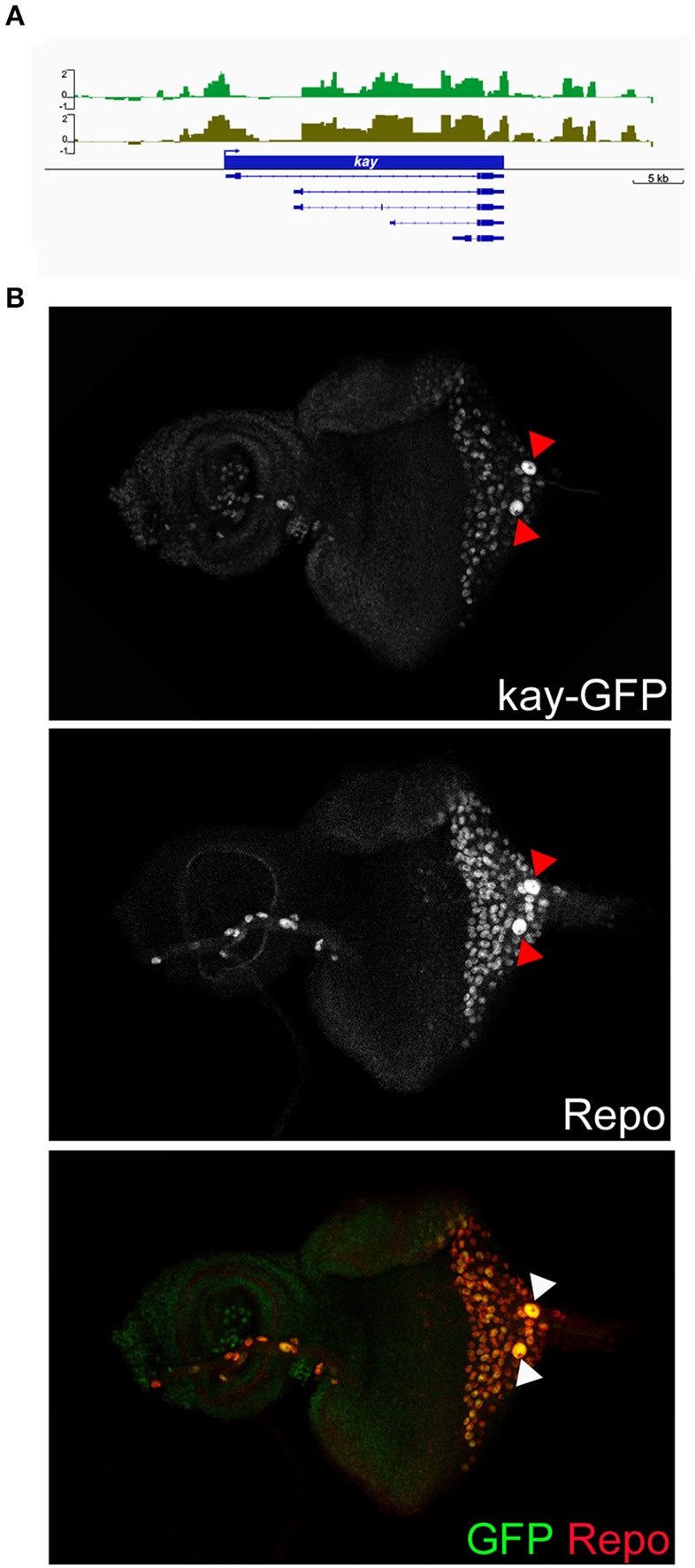
Characterization of Dam-Pol II-bound *kay*. **(A)** Dam-Pol II occupancy in the *kay* locus. Both biological replicates are shown in light and dark green. Scale bars on the *y*-axis represent the log_2_ ratio change between Dam-Pol II and Dam samples. **(B)** Expression of GFP-tagged Kay protein. Larval eye imaginal disc stained for Kay protein (GFP staining, green) and glial nuclei (Repo staining, red). Red and white arrowheads indicate the carpet nuclei.

The over-represented GO terms of the carpet glia transcriptome suggest that the majority of genes in the carpet glia function in cellular activities related to its dynamic morphological changes in the developing eye. We first examined genes encoding transcription factors (TFs) to identify upstream regulators of carpet cell morphology. We categorized genes whose protein products annotated as TFs with GO term annotations related to cell migration, dorsal closure, and wound repair into one group (Table S2). Table 2 lists the 10 genes showing the highest expression in this TF group. Encouragingly, the *apt* gene, known to be expressed mainly in the PG and weakly in the SPG (Sasse and Klämbt, 2016), was included in the group, demonstrating the sensitivity of the Dam-Pol II-bound transcriptome of carpet glia. The gene *kayak* (*kay*) encodes the *Drosophila* Fos and functions downstream of several signaling pathways, including EGF and JNK signaling (Ciapponi et al., 2001). The role of Kay in cell morphology has long been characterized. It is required for embryonic dorsal closure and epithelial cell fusion of imaginal discs during pupal development (Riesgo-Escovar and Hafen, 1997; Zeitlinger et al., 1997; Ciapponi et al., 2001). The *kay* locus exhibits significant Dam-Pol II occupancy (Figure 3A). We examined its expression using a readily available fly line whose Kay protein is endogenously tagged (Nagarkar-Jaiswal et al., 2015). Co-staining of the protein-tag GFP and the glial nuclei marker Repo demonstrates Kay expression in the nuclei of carpet glia (Figure 3B), which is consistent with its TF activity. Kay is also expressed in a subset of RBGs (Figure 3B). The expression of Kay in the carpet glia, although not exclusively, verifies the quality of the Dam-Pol II binding data. To further investigate the role of Kay in the carpet glia, we silenced *kay* expression using two independent *kay*^*dsRNA*^ constructs with *C135-Gal4* and *UAS-mCD8GFP* labeling the membrane of carpet glia. However, we did not observe any defects in the carpet cell shape (Figure S2).

**Table 2:** Transcription factors involved in cell movement.

**Gene name (Symbol)**	**Average log_**2**_ FC**	**GOTERM_Biological Process_DIRECT**	**GOTERM_Molecular Function_DIRECT**
*knirps (kni)*	1.46	GO:0007427~epithelial cell migration, open tracheal system	GO:0001078~transcriptional repressor activity, RNA polymerase II core promoter proximal region sequence-specific binding
*anterior open* (*aop*)	1.10	GO:0007298~border follicle cell migration	GO:0003705~transcription factor activity, RNA polymerase II distal enhancer sequence-specific binding
*knirps-like* (*knrl*)	0.98	GO:0007427~epithelial cell migration, open tracheal system	GO:0003700~transcription factor activity, sequence-specific DNA binding
*tramtrack* (*ttk*)	0.98	GO:0007298~border follicle cell migration	GO:0001078~transcriptional repressor activity, RNA polymerase II core promoter proximal region sequence-specific binding
*bunched* (*bun*)	0.97	GO:0007297~ovarian follicle cell migration	GO:0003700~transcription factor activity, sequence-specific DNA binding
*kayak* (*kay*)	0.90	GO:0007298~border follicle cell migration GO:0007391~dorsal closure GO:0009611~response to wounding	GO:0003700~transcription factor activity, sequence-specific DNA binding
*ultraspiracle* (*usp*)	0.87	GO:0007298~border follicle cell migration	GO:0001077~transcriptional activator activity, RNA polymerase II core promoter proximal region sequence-specific binding
*Ecdysone receptor* (*EcR*)	0.71	GO:0006911~phagocytosis, engulfment GO:0007155~cell adhesion GO:0007298~border follicle cell migration	GO:0001077~transcriptional activator activity, RNA polymerase II core promoter proximal region sequence-specific binding
*yorkie* (*yki*)	0.52	GO:0007298~border follicle cell migration	GO:0003713~transcription coactivator activity
*apontic* (*apt*)	0.51	GO:0007298~border follicle cell migration	GO:0003700~transcription factor activity, sequence-specific DNA binding

*For simplicity, no more than three GO terms for each gene are listed. For details, please see Table S2*.

**Figure 4:**
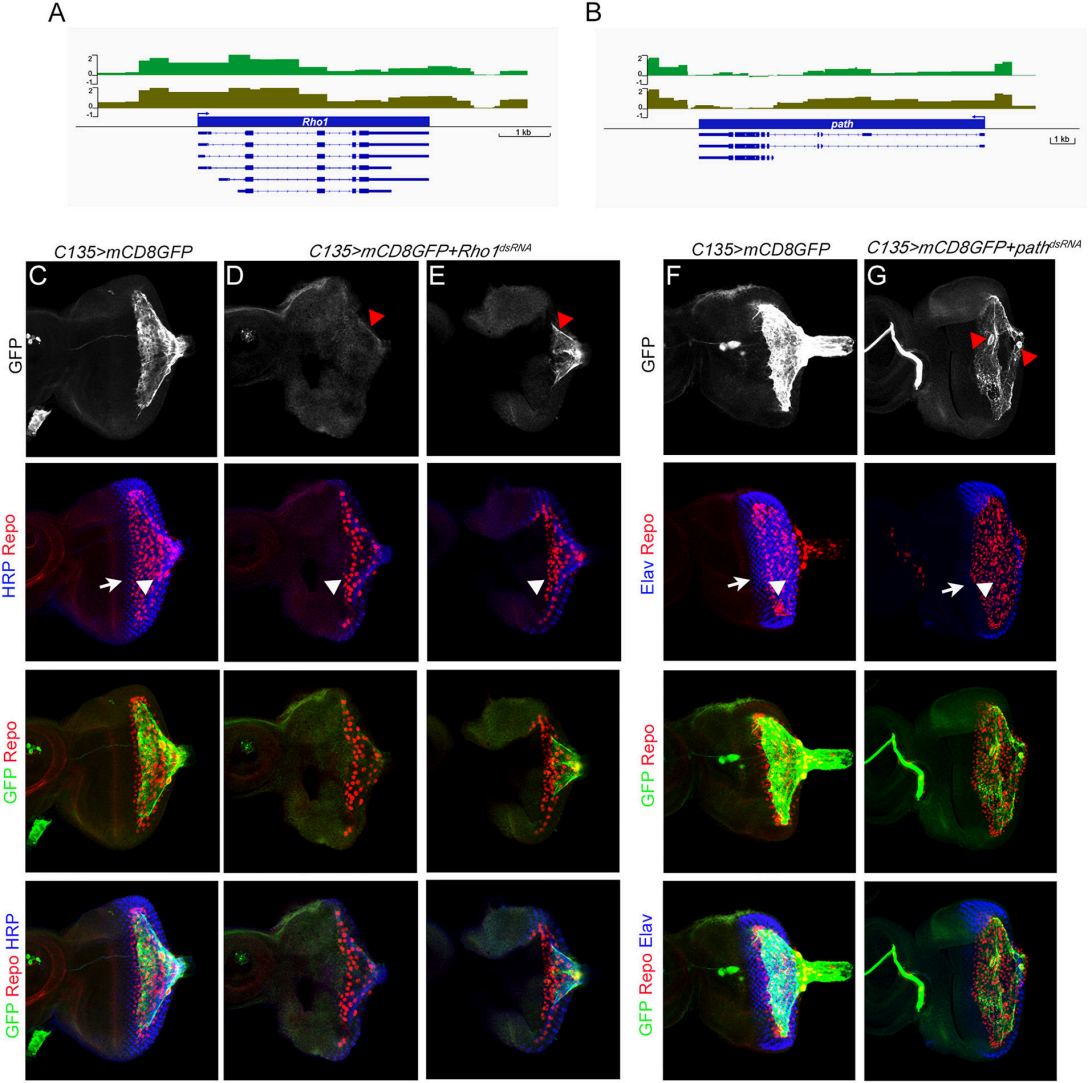
Characterization of Dam-Pol II-bound genes: *Rho1* and *path*. **(A,B)** Dam-Pol II occupancy in the *Rho1* **(A)** *and path* **(B)** loci. Both biological replicates are shown in light and dark green. Scale bars on the *y*-axis represent the log_2_ ratio change between Dam-Pol II and Dam samples. **(C–E)** All discs stained for the presence of carpet glia membrane (GFP staining, green), glial nuclei (Repo staining, red), and neuronal membrane (HRP staining, blue). **(F,G)** All discs stained for the presence of carpet glia membrane (GFP staining, green), glial nuclei (Repo staining, red), and differentiated PRs (Elav staining, blue). **(C–G)** The first row shows the membrane of carpet glia stained with GFP. Red arrowheads in **(D,E)** indicate the remaining membrane, and red arrowheads in **(G)** indicate the nuclei of carpet glia. The second row shows the relative position of the front of the PRs and that of the glia stained with HRP **(C–E)**/Elav **(F,G)** and Repo and indicated by white arrows and arrowheads, respectively. The third row shows the merged staining of Repo and GFP for the relative position between RBGs and the front of the carpet glial membrane, and the fourth row shows images merged with all staining. All images are z-projection of confocal sections.

To directly probe molecules that may be involved in regulating carpet cell morphology, we categorized genes with functional annotations in any of the three GO term fields related to cytoskeleton, actin, microtubules, and cell adhesion into a second group (Table S2). Table 3 lists 10 highly expressed genes in the cytoskeleton group. Among them, the *Rho1* gene was annotated with the most GO terms associated with cytoskeleton organization, cell polarity, and cell movement during development and wounding. Rho1 is a GTPase and a major regulator of the actin cytoskeleton in an array of developmental processes, including germ band extension, myoblast fusion, and dorsal closure during embryogenesis (Barrett et al., 1997; Lu and Settleman, 1999; Jacinto et al., 2002; Barmchi et al., 2005; Kim et al., 2015); morphogenesis of imaginal discs (Larson et al., 2008; Manning et al., 2013); migration of hemocytes, follicle border cells, and glia (Sepp and Auld, 2003; Stramer et al., 2005; Bastock and Strutt, 2007); and wound repair (Abreu-Blanco et al., 2014). It has been shown that Rho1 acts downstream of the GPCR Trapped in endoderm 1 (Tre1) and Smog to regulate, respectively, embryonic germ cell migration and mesoderm invagination during gastrulation (Kunwar et al., 2008; Kerridge et al., 2016). Since the subperineurial glia-specific GPCRs Loco and Moody regulate cell growth and shape during the mesenchymal to epithelial transition when the migratory SPGs form a squamous, secondary epithelium enveloping the brain during embryogenesis (Schwabe et al., 2017), it is of significance to examine the role of Rho1, as a potential effector downstream of Loco and Moody, in regulating carpet cell morphology. We knocked down *Rho1* expression using two independent *Rho1*^*dsRNA*^ constructs with *C135-Gal4* and *UAS-mCD8GFP* labeling the membrane of carpet glia. Both *Rho1*^*dsRNA*^ alleles displayed defects in forming intact plasma membrane with different degrees of severity. *Rho1* expression knockdown resulted in either no detectable GFP staining or only a trace in the severely affected eye discs (Figure 4D, 6/11 discs), and the GFP-positive membrane of carpet glia in the less affected eye discs showed negligible extension toward the anterior and remained in the posterior center of the eye disc (Figure 4E, 5/11 discs), indicating that the cytoskeleton of carpet glia was disrupted. Moreover, we observed that the RBGs marked by the glial nuclei marker Repo moved farther anteriorly and reached the differentiating PRs in all membrane-defective discs, compared with the control discs of *C135-Gal4* driving only *UAS-mCD8GFP* (Figures 4C–E). The overmigratory glial cell phenotype resulting from membrane-defective carpet glia is consistent with the proposed function of carpet glia in retaining RBG migration (Silies et al., 2007).

**Table 3:** Molecules involved in cytoskeleton functions.

**Gene name**	**Average log_**2**_ FC**	**GOTERM_Biological Process_DIRECT**	**GOTERM_Molecular Function_DIRECT**
*alpha-Tubulin at 84B* (*alphaTub84B*)	2.28	GO:0007017~microtubule-based process	GO:0003924~GTPase activity GO:0005200~structural constituent of cytoskeleton GO:0005525~GTP binding
*chickadee* (*chic*)	1.79	GO:0000902~cell morphogenesis GO:0007015~actin filament organization	GO:0003779~actin binding GO:0003785~actin monomer binding
*Actin 5C* (*Act5C*)	1.59	GO:0007010~cytoskeleton organization	GO:0005200~structural constituent of cytoskeleton GO:0005524~ATP binding
*Dynein light chain 90F* (*Dlc90F*)	1.51	GO:0007018~microtubule-based movement	GO:0042623~ATPase activity coupled GO:0045505~dynein intermediate chain binding
*Moesin* (*Moe*)	1.43	GO:0002009~morphogenesis of an epithelium GO:0007010~cytoskeleton organization	GO:0003779~actin binding GO:0008017~microtubule binding GO:0008092~cytoskeletal protein binding
*Actin 42A* (*Act42A*)	1.38	GO:0006909~phagocytosis GO:0007010~cytoskeleton organization	GO:0005200~structural constituent of cytoskeleton GO:0005524~ATP binding
*14-3-3epsilon*	1.29	GO:0008103~oocyte microtubule cytoskeleton polarization	GO:0019904~protein domain specific binding
*twinstar* (*tsr*)	1.24	GO:0001736~establishment of planar polarity GO:0007015~actin filament organization	GO:0003779~actin binding
*Rho1*	1.23	GO:0007395~dorsal closure, spreading of leading edge cells GO:0008347~glial cell migration GO:0051493~regulation of cytoskeleton organization	GO:0003924~GTPase activity GO:0019900~kinase binding
*Cytoplasmic linker protein 190* (*CLIP-190*)	1.23	GO:0007017~microtubule-based process GO:0007349~cellularization	GO:0003779~actin binding GO:0008017~microtubule binding GO:0070854~myosin VI heavy chain binding

*For simplicity, no more than three GO terms for each gene are listed. For details, please see Table S2*.

In addition, we examined the role of the *path* gene where the *C135-Gal4* insertion is located. The *path* gene encodes an amino acid transmembrane transporter that localizes to the cell surface and endolysosomal compartments and is required for the growth of neurons with large dendrite arbors during embryogenesis (Lin et al., 2015). We silenced *path* expression with a *path*^*dsRNA*^ construct driven by *C135-Gal4* and *UAS-mCD8GFP* labeling the membrane of carpet glia. The eye discs with *path* knockdown exhibited breakage between the two carpet cells (Figure 4G, 4/6 discs). The broken opening between two carpet cells was always in the posterior center of the eye disc. Intriguingly, the relative position of the two carpet cells in the eye disc also changed (Figure 4G, 3/4 discs). It has been reported that carpet glial cells obey the dorsal–ventral equator of the compound eye, and each carpet cell occupies half of the eye disc (Silies et al., 2007). We found that two carpet glial cells became apically to basally positioned in the eye discs with *path* knockdown, and one carpet cell covered the entire eye disc (Figure 4G). Despite the breakage between the two carpet cells, the glial cell migratory pace was maintained. Glial cells were two to three cells posterior to the differentiating PRs compared with the control (Figures 4G,F). These results suggest that *path* affects carpet cell morphology by regulating the intercellular contacts between two carpet cells.

Since we uncovered several characteristic BBB genes in the carpet glia when comparing the transcriptome of carpet glia to those of the adult *Drosophila* surface glia and the mouse BBB, we explored whether the development of carpet glia affects the integrity of the BBB. We injected fluorescently labeled dextran into the abdomen of *Rho1-*knockdown flies driven by *C135-Gal4*, whose carpet glia were malformed with defective plasma membrane, and examined dye penetration in adult eyes. Little to no fluorescence was observed in the eyes of the control flies carrying *C135-Gal4* driving *UAS-mCD8GFP*, whereas considerable fluorescence was detected in the eyes of the *Rho1*-knockdown flies (Figures S3A,B), indicating that the BBB of the *Rho1*-knockdown flies was impaired. *C135-Gal4* drives expression in the carpet glia as well as the surface glia surrounding the brain during larval development (Figure S3C), and no Gal4 activity is detected in the adult brain (Yeh et al., 2018). Therefore, BBB leakage upon *C135-Gal4* driven *Rho1* knockdown, at least in part, results from the defective carpet glia morphogenesis during larval development.

### Components of Signaling Pathways Identified in the Carpet Glia Transcriptome

**Figure 5:**
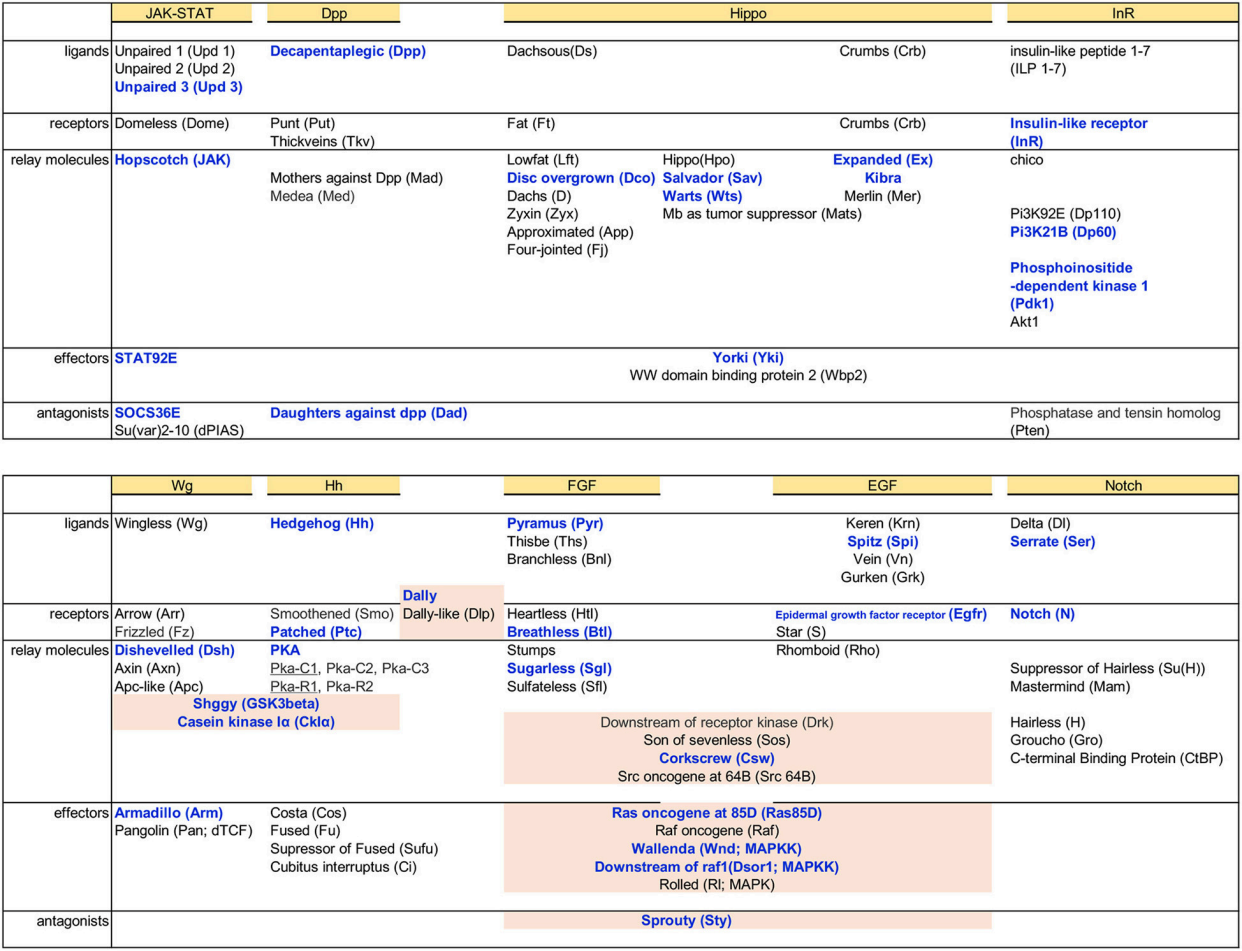
Dam-Pol II binding to components of signaling pathways. Basic components of each signaling pathway, from the top ligands, receptors, signal relay molecules, effectors, and antagonists, are listed. Genes bound by Dam-Pol II are shown in bold blue. Components shared between pathways are highlighted in light pink.

In addition to TFs and genes functioning in cytoskeleton organization, we also examined Dam-Pol II binding to components of signaling pathways. In the developing eye, RBGs migrate along the carpet glia, get contact with differentiated PRs, and enwrap PR axons for their projection into the brain (Rangarajan et al., 1999; Hummel et al., 2002; Silies et al., 2007). The entire process requires intensive cell–cell interactions. It is highly likely that carpet glial cells send and receive signals for intercellular communications to regulate retinal glial migration. We found Dam-Pol II binding to the components of several signaling pathways, including JAK-STAT, Decapentaplegic (Dpp), insulin-like receptor (InR), Notch (N), Wingless (Wg), Hedgehog (Hh), Hippo, epidermal growth factor (EGF), and fibroblast growth factor (FGF), which are reiteratively utilized in various developmental processes (Garofalo, 2002; Shilo, 2005; Arbouzova and Zeidler, 2006; Andersson et al., 2011; Bejsovec, 2013; Briscoe and Thérond, 2013; Muha and Müller, 2013; Hamaratoglu et al., 2014; Pfleger, 2017; Figure 5). Multiple genes, which encode members of FGF and EGF signaling pathways, from upstream ligands to downstream effectors, were bound by Dam-Pol II. The role of FGF signaling in coordinating glial proliferation and migration in the developing eye has been characterized (Franzdóttir et al., 2009). Two FGF8-like ligands, Pyramus (Pyr), and Thisebe (Ths), are expressed in distinct cell populations. Pyr is expressed in glia and cells anterior to the morphogenetic furrow, whereas Ths is expressed in PRs (Franzdóttir et al., 2009). Our carpet glia transcriptome is consistent with the previous study. Only *pyr*, not *ths*, was bound by Dam-Pol II (Figure S1D).

**Figure 6:**
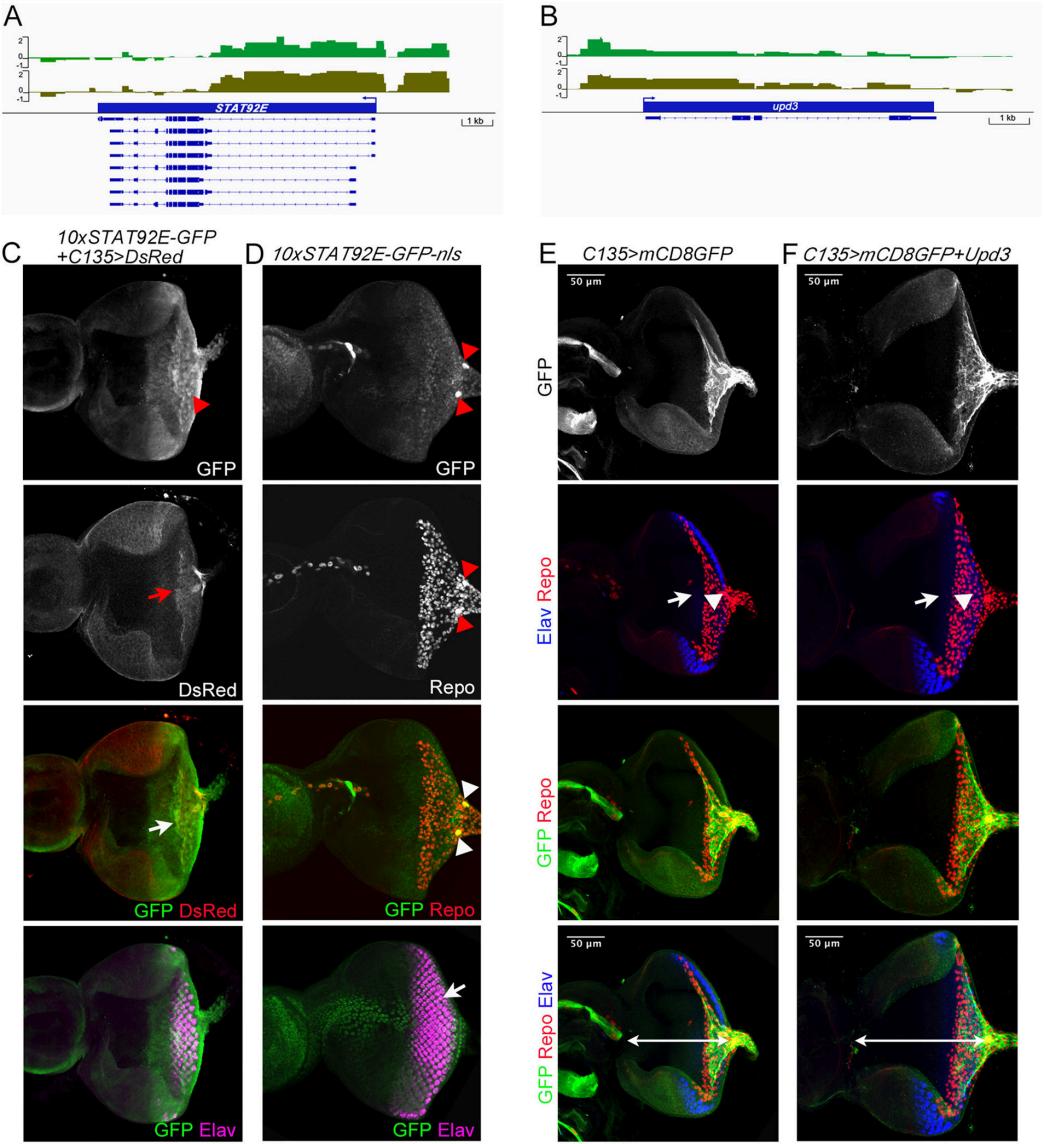
Characterization of Dam-Pol II-bound genes: *STAT92E* and *upd3*. **(A,B)** Dam-Pol II occupancy in the *STAT92E* **(A)** and *upd3* **(B)** loci. Both biological replicates are shown in light and dark green. Scale bars on the *y*-axis represent the log_2_ ratio change between Dam-Pol II and Dam samples. **(C)** A STAT92E activity reporter containing 10 STAT92E binding sites (10× STAT92E-GFP) was crossed with *C135-Gal4* driving *UAS-DsRed* labeling the membrane of carpet glia. GFP is expressed in the cytoplasm. The disc shown is a z-projection image and stained for the presence of GFP (green), DsRed (red), and Elav labeling differentiated PRs (magenta). The red arrowhead indicates the GFP punctate. The red arrow indicates the carpet glia membrane, and the white arrow indicates the colocalization of GFP and DsRed signals. **(D)** A modified STAT92E activity reporter whose GFP reporter is nucleus localized (10× STAT92E-GFP-nls). The disc shown is stained for the presence of GFP (green), Repo (red), and Elav (magenta). The merged GFP and Repo image is a z-projection of basal focal planes, and the merged GFP and Elav image is the same eye disc with a z-projection of apical focal planes. Red and white arrowheads indicate the carpet nuclei. The white arrow indicates the colocalization of GFP and Elav signals. **(E,F)** All discs stained for the presence of carpet glia membrane (GFP staining, green), glial nuclei (Repo staining, red), and differentiated PRs (Elav staining, blue). Upd3-expressing eye discs **(F)** and control discs **(E)** at similar developmental stages (mid-third instar) are compared for disc size, which is measured by the length of the disc midline indicated by the white line. Upd3-expressing discs: 153 ± 2.7 μm, *n* = 10; control discs: 126 ± 3.6 μm, *n* = 8 (mean ± SD, *n* = disc number, *p* < 0.001, Student's *t*-test). Rows of images are arranged as Figure 4. White arrows and arrowheads in both **(E,F)** indicate the Elav-stained PRs and the Repo-stained glial nuclei, respectively.

The ability of carpet glia to regulate RBG migration is mainly mediated through their extensive membrane (Silies et al., 2007). It is of interest to explore whether the signaling pathways identified in the carpet glia transcriptome are involved in regulating carpet cell morphology and thereby affect RBG migration. JAK-STAT signaling is of particular interest because of the expression of the Unpaired (Upd) ligand in the midline of the posterior eye disc margin, where it coincides with the bilaterally located nuclei of carpet glia (Tsai and Sun, 2004). We observed Dam-Pol II binding to the genes encoding the ligand Unpaired 3 (Upd3), the tyrosine kinase Hopscotch (Hop/JAK), the signal transducer and TF STAT92E, and also the signaling antagonist SOCS36E. Interestingly, there was no Pol II binding to the receptor gene *domeless* (*dome*; Figures 6A,B). We assessed whether JAK-STAT signaling is active in the carpet glia. Immunostaining of the eye discs carrying a STAT92E activity reporter, which contains 10 STAT92E binding sites linked to a GFP reporter gene (Bach et al., 2007), showed punctate GFP expression in the posterior part of the eye disc, which overlaps with the carpet glia (Figure 6C). We further examined the expression of a modified STAT92E activity reporter whose GFP is nucleus localized (Tsai et al., 2015) and found GFP expression in the carpet nuclei (Figure 6D). In addition, active JAK-STAT signaling was also found in the migratory RBGs and the PRs in the posterior part of the eye disc (Figure 6D). Conversely, we overexpressed Upd3 using *C135-Gal4* to examine its effect on carpet cell morphology when carpet cells act as signal-sending cells. At similar developmental stages, the Upd3-expressing eye discs were generally larger than the control discs of *C135-Gal4* driving only *UAS-mCD8GFP* (Figures 6E,F). It has been reported that Upd3 promotes cell proliferation in the developing eye (Wang et al., 2014). The overgrowth of Upd3-expressing eye discs suggests that the Upd3 protein expressed in carpet glia can be secreted to act as cell proliferation signal. Nevertheless, we observed that upon Upd3 overexpression, carpet glia formed normally with anteriorly extending membrane (Figures 6E,F). Altogether, these results suggest that the carpet glia can both receive JAK-STAT signaling and secrete Upd3 to regulate neighboring cells.

## Discussion

Here, we present the transcriptional profile of carpet glia, a specific SPG type in the developing eye, through targeted DamID-seq. This method takes advantage of the Gal4-UAS system and enables gene expression profiling of a specific cell population without cell isolation. Because we expressed Dam-Pol II specifically using the *C135-Gal4* driver and the isolated eye disc contains only two giant carpet cells with *C135-Gal4* expression, our results provide a very clean and specific analysis of the two carpet glial cells. It has been shown that Dam-Pol II binding recapitulates endogenous Pol II binding (Southall et al., 2013), and the coverage of genes expressed in the carpet glia, including *moody, spin*, and *pyr*, in our Dam-Pol II binding profile of the carpet glia validates the sensitivity of this method and the quality of our data.

We found that numerous genes with significant Dam-Pol II occupancy have been characterized in cellular activities that undergo changes in cell morphology, such as wing imaginal disc morphogenesis, border cell migration during oogenesis, and dorsal closure during embryogenesis. These cellular activities agree with the feature of the carpet cells that migrate into and extend along the developing eye. To explore the roles of these Dam-Pol II–bound genes, we selected several genes with distinct functions, including the genes encoding TFs, genes acting as cytoskeleton regulators, genes regulating growth, and components of signaling pathways for intercellular communication. We silenced the expression of the selected genes specifically in the carpet glia by RNAi and observed the effect on carpet cell morphology and its function of restraining RBG migration. Reduced expression of Rho1 results in severe disruption of carpet cell morphology and impairs the ability of carpet glia to restrict RBG migration. The adult BBB is also compromised to allow dye leakage, probably a consequence of the defective carpet glial morphology. Carpet cells exhibit unique morphological features that the cells are large, flat, and thin. They possess extensive plasma membrane that is a result of continuous extension to the anterior of the eye field in a pace that follows PR differentiation. During anterior extension, the plasma membrane of carpet glia undergoes constant protrusion–retraction cycles as the protrusion of plasma membrane can be clearly observed by immunostaining and by live imaging (Tsao et al., 2016). It has been shown that Rho1 drives actin polymerization at the leading edge and acts as a pacemaker of protrusion–retraction cycles (Machacek et al., 2009; Tkachenko et al., 2011). A recent study shows that the Moody GPCR signaling pathway controls protrusive activity and stability at the leading edge of subperineurial glia when the cells transit from the migratory state to form an epithelium enwrapping the embryonic brain (Schwabe et al., 2017). It has been reported that Rho1 is identified as an important downstream effector of Moody signaling during the process (Schwabe et al., 2017). Our data of disrupted carpet cell morphology upon the reduction of Rho1 expression are in line with this study and suggest that Rho1 is downstream of the subperineurial glia-specific Moody pathway in the carpet glia. Moreover, it has been demonstrated that Rho1 regulates the formation of *Drosophila* E-cadherin (DE-cadherin)-containing, Rab11-positive recycling endosomes in *Drosophila* pupal eye epithelium and thereby influences adherens junction remodeling (Yashiro et al., 2014). We identified a significant number of genes functioning in endocytosis in the carpet glia transcriptome, including various Rab proteins and several components of adherens junctions (Figure 2 and Table S2), suggesting a possible mechanism that Rho1 regulates carpet cell morphology through endocytosis components. In addition, Spin, a late endosomal/lysosomal protein, is required for the growth of carpet glia (Yuva-Aydemir et al., 2011). In *spin* mutants, carpet cell shape is severely affected (Yuva-Aydemir et al., 2011), providing evidence that components in endocytosis function in carpet glia morphogenesis. Therefore, our carpet glia transcriptome covers the whole spectrum of the morphology regulators from the upstream GPCR Loco and Moody, the significant effector Rho1 to the downstream endocytosis proteins, and presents a potential molecular mechanism underlying the unique morphological features and cellular activities of carpet glia.

The silencing of *path*, although not disrupting carpet cell morphology as severely as that of *Rho1*, breaks the connection between two carpet cells. The *Drosophila* Path protein is closely related to the vertebrate solute carrier (SLC) transporter SLC36A4 (Lin et al., 2015). We discovered several genes with transporter activity in the carpet glia transcriptome. One of them, *CG3168*, is closely related to the mouse SLC2A1, an SLC transporter shuttling glucose between the blood and the brain (Boado and Pardridge, 1990; Pardridge et al., 1990). It has been proposed that Path may function as a transporter-like protein with a receptor activity (transceptor) that responds to metabolites and triggers downstream signaling (Lin et al., 2015). The breakage between carpet glia upon *path* knockdown suggests that this specific SLC transporter in the carpet glia likely functions in communication between two carpet cells. The carpet glia transcriptome also includes several components of insulin signaling pathway (Figure 5). It will be intriguing to explore the possibility of Path as a transceptor and its potential crosstalk with insulin signaling pathway.

Kay, the *Drosophila* Fos, is the downstream effector of JNK signaling during embryonic dorsal closure and acts with its heterodimer partner, Jun-related antigen (Jra, the *Drosophila* Jun), in a nonredundant manner (Zeitlinger et al., 1997; Ciapponi et al., 2001). The *kay* expression detected in carpet glia encourages us to examine its function in carpet cell morphology despite no Dam-Pol II binding observed to the upstream components of JNK signaling, including the MAPKKK Misshapen (Msn), MAPKK Hemipterous (Hep), MAPK Basket (Bsk), or its partner Jra. Reduced Kay expression in the carpet glia shows no effect on carpet glial cell morphology, suggesting that Kay in the carpet glia may be involved in other functions, such as cell cycle regulation (Hyun et al., 2006). This may hold true for the functions of other signaling pathways in the carpet glia as well.

Several components of various signaling pathways are bound by Dam-Pol II in the carpet glia transcriptome. We examined the activity of JAK-STAT signaling in regulating carpet cell morphology, which subsequently affects RBG migration. We overexpressed Upd3 in the carpet glia to test whether there is a cell non-autonomous effect that Upd3 is received by other retinal glial cells or neurons on carpet cell morphology. The loss-of-function strategy of knocking down *upd3* was not taken since the effect of reducing Upd3 expression is likely to be rescued by other Upd3-secreting cells located in the posterior midline of the eye disc (Wang et al., 2014). Upd3 overexpression caused overgrowth in eye disc, suggesting that Upd3 in the carpet glia can be secreted and may induce cell proliferation in the eye disc. Upd3 overexpression had no effect on carpet cell morphology, suggesting that carpet glia morphogenesis is not regulated by Upd3 signal. Conversely, we found that STAT92E is active in the carpet glia. However, we also found the active STAT92E signals in other glial cells as well as some PRs. Since the receptor Dome is not identified in the carpet glia transcriptome, there might be integration of other signalings to activate STAT92E in the carpet glia. It has been shown that the receptor tyrosine kinase Torso (Tor), when hyperactivated, can activate STAT92E (Li et al., 2003). Although Tor has no significant Dam-Pol II binding in the carpet glia transcriptome, we identified several components of RTK signaling pathways such as EGF and FGF signaling pathways. It remains to be determined whether STAT92E can be activated by the RTK signaling pathways in the carpet glia. It has also been shown that the JAK-STAT pathway can be activated by the secreted peptide Vago in a Dome-independent manner (Paradkar et al., 2012). The putative Vago receptor has not yet been identified. Our carpet glia transcriptome analysis may provide clues to the non-canonical JAK-STAT activation. It also remains to be determined what is the specific cellular function of the active STAT92E signaling in the carpet glia.

In this study, we focused on genes involved in regulating cell morphology based on the results of GO term analysis showing genes enriched in cellular activities of cytoskeleton rearrangement. Numerous genes identified in the carpet glia transcriptome are yet to be studied, including genes that are not included in the over-represented GO terms shown in Figure 2. These “under-represented” genes are listed in Table S2. They are genes with diverse functions, and many of these genes were previously uncharacterized. Therefore, the validated carpet glia transcriptome provides ample gene information for further studying the functions of carpet glia in the developing eye, for example, the mechanisms regulating the initial contact, migration, and final detachment of migratory RBGs from the cell surface of carpet glia. Furthermore, obtaining a carpet glia-specific gene set would benefit the study of cellular interaction between the carpet glia and other retinal glial cell types. Such a carpet glia-specific gene set can be achieved in the future through the validated targeted DamID-seq method. Expression profiles of specific cell types, including wrapping glia and migratory RBG, can be constructed and compared against the carpet glia transcriptome. A recent study also used the targeted DamID-seq method and profiled the genes expressed in two cell types of the *Drosophila* midgut, intestinal stem cells/differentiating enteroblast progenitors (ISCs/EBs), and absorptive enterocytes (ECs; Doupé et al., 2018). Following more studies conducted by targeted DamID-seq, transcriptomes of various cell types will be available. Genes exclusively expressed in individual cell types can be sorted out by combinatorial comparisons between transcriptomes of distinct cell types to establish cell-type-specific gene sets.

GO term analyses of the 456 genes expressed in the transcriptomes of both the carpet glia and adult surface glia demonstrate that several cellular processes and components are common between the carpet glia and adult BBB, such as cell morphogenesis and movement, microtubule-associated complex, and the endomembrane system (Table S3), suggesting that the carpet glia's molecular signature of morphology regulators is retained, at least in part, in the adult BBB. Further comparison of the genes common to the carpet glia and adult surface glia with the mouse BBB transcriptome reveals several *Drosophila* genes whose predicted mouse orthologs encode characteristic BBB proteins (Table 1 and Table S3). Therefore, using carpet glia as a platform is comparable to the adult *Drosophila* BBB. *C135-Gal4-*driven *Rho1* knockdown impaired the retinal part of the BBB, i.e., the blood–retina barrier (BRB). *C135-Gal4* drives expression in both developing carpet glia and the surface glia surrounding the larval brain, and no Gal4 activity is detected in the adult brain. Because the brain BBB is formed primarily during late embryogenesis (Beckervordersandforth et al., 2008; von Hilchen et al., 2013), the effect of BRB impairment likely results from the defective carpet glia. The *Drosophila* BRB morphologically resembles the mammalian BBB in that they both enclose the organ being insulated (Carlson et al., 2000). An intact BRB is established when the meeting point of the optic stalk and the brain is closed during pupal development (Carlson et al., 1998, 2000). At similar pupal stages, the carpet glial cells migrate back to the brain and lie underneath the lamina neuropil (Edwards et al., 2012). The BRB leakage, caused by knocking down *Rho1* in only the larval stages when carpet glial cells are developing, coincides with the migratory behavior of carpet glia back to the brain and the developmental timing of BRB closure, suggesting that the development of carpet glia may be involved in the formation of a functional BRB.

Overall, we provide a validated carpet glia transcriptome, which has identified several characteristic BBB genes common to the adult *Drosophila* surface glia and the mouse BBB, as well as cell morphology regulators illustrating the unique features of carpet glia. This single-cell-type transcriptome is a resource for enhancing the further understanding of this particular cell type as well as the entire tissue.

## Data Availability

The DamID-sequencing reads have been deposited in ArrayExpress under the accession number E-MTAB-7475.

## Author Contributions

Y-HL developed the concepts, performed experiments, analyzed the results, and wrote the manuscript. T-YH, W-HW, and Y-ML performed experiments and analyzed the results. S-JH and TL performed computational analyses of DamID-seq data.

### Conflict of Interest Statement

The authors declare that the research was conducted in the absence of any commercial or financial relationships that could be construed as a potential conflict of interest.

## Supplementary Material

Figure S1 Quality assessment of the Dam-Pol II binding data. **(A)** Reproducibility between two biological replicates. The intensity of each Dam-Pol II to Dam-only binding peak at the same genomic location is indicated by red crosses. **(B–D)** Positive controls: Dam-Pol II occupancy of the genes expressed in the carpet glia. **(B)** *moody* locus, **(C)** *spinster* (*spin*) locus, and **(D)** *pyramus* (*pyr*) locus. Both biological replicates are shown in light and dark green. Scale bars on the *y*-axis represent the log_2_ ratio change between Dam-Pol II and Dam samples.

Figure S2 *kay* knockdown has no effect on carpet cell morphology. **(A,B)** All discs stained for the presence of carpet glia membrane (GFP staining, green), glial nuclei (Repo staining, red), and differentiated PRs (Elav staining, blue). The first row shows the membrane of carpet glia stained with GFP. The second row shows the relative position of the front of the PRs and that of the glia stained with Repo. White arrows and arrowheads in both A and B indicate Elav-stained PRs and Repo-stained glial cells, respectively. The third row shows the merged staining of Repo and GFP for the relative position between RBGs and the front of the carpet membrane, and the fourth row shows images merging all staining.

Figure S3 Blood–retina barrier permeability assay. **(A)** Confocal images of the heads of the control (upper) and *Rho1* knockdown (lower) flies. **(B)** Quantification of the fluorescence intensity detected in each eye. *Rho1* knockdown discs: *n* = 18; control discs: *n* = 11 (*p* < 0.0001, Student's *t*-test). **(C)** Expression of *C135-Gal4* driving *UAS-mCD8GFP* in the larval brain and eye disc. The cell membrane of surface glia surrounding the larval brain, indicated by red arrowheads, and that of the carpet glia, indicated by white arrow, is marked by mCD8GFP (green). Glial nuclei are labeled by Repo (red).

Table S1 Transcriptome of carpet glia. All genes meeting the criteria of the average log_2_ ratio of Dam-Pol II to Dam >0.5 and an FDR < 0.01 are listed for both biological replicates.

Table S2 Protein-coding genes used in GO term analysis of the carpet glia transcriptome. Genes are listed as in groups: (1) TFs, (2) components of signaling pathways, (3) cytoskeleton, and (4) all others. GO terms of each gene analyzed by DAVID are listed. Genes are also labeled as included or not included in the enriched GO terms described in Figure 2.

Table S3 Genes common to the transcriptomes of carpet glia, adult *Drosophila* BBB, and the mouse BBB. Common genes identified in the transcriptomes of carpet glia (this study) and the adult *Drosophila* surface glia (DeSalvo et al., 2014) and the mouse orthologs of these genes identified by comparing with the mouse BBB transcriptome (Daneman et al., 2010) are listed.
