# Supplementary figures and images for: Expressional Profiling of Carpet Glia in the Developing Drosophila Eye Reveals Its Molecular Signature of Morphology Regulators
Source: Front Neurosci. 2019 Mar 29;13:244. doi: 10.3389/fnins.2019.00244 (PMC6449730; doi:10.3389/fnins.2019.00244)

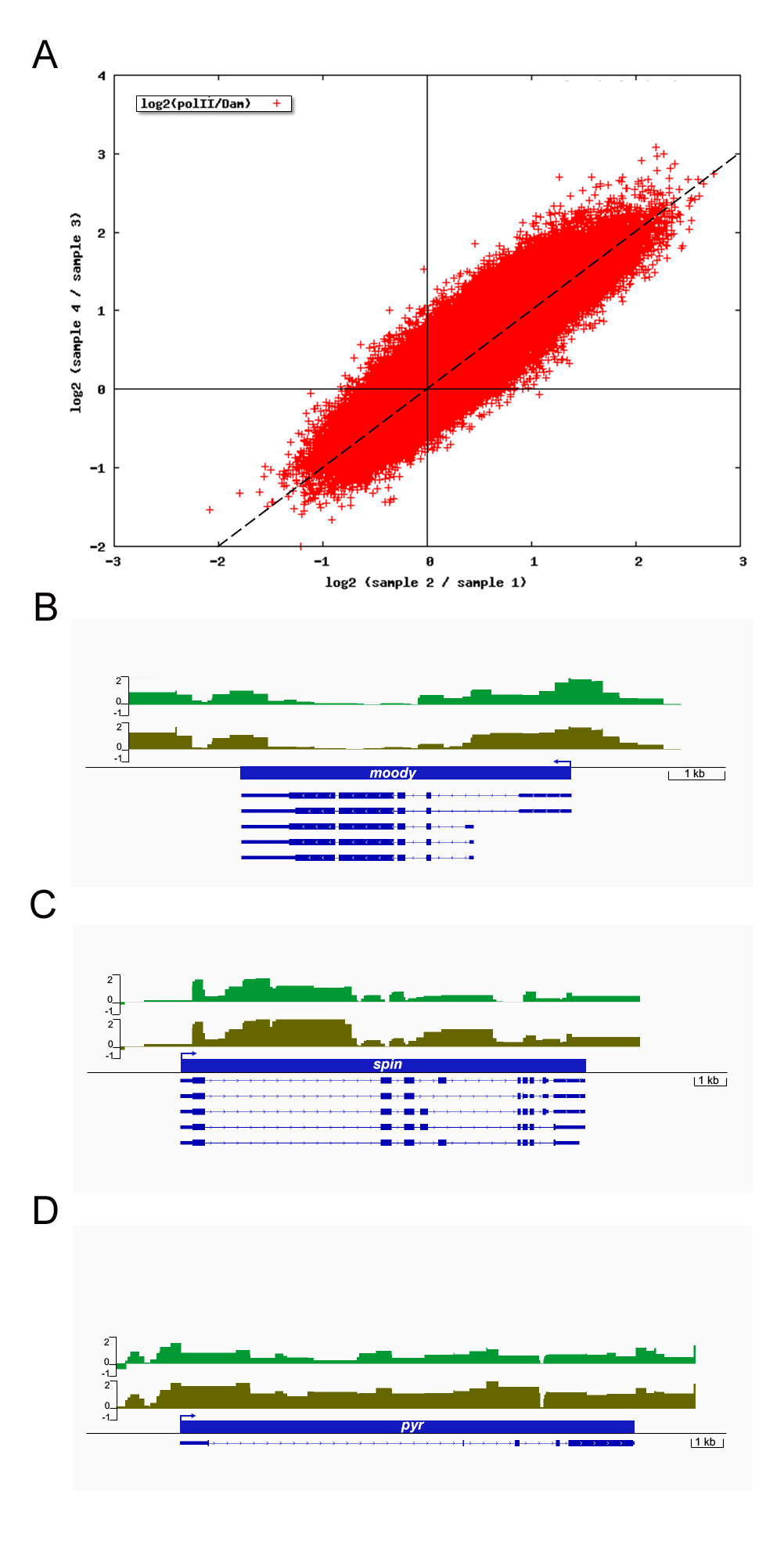

Supplement: Figure S1 — Quality assessment of the Dam-Pol II binding data. (A) Reproducibility between two biological replicates. The intensity of each Dam-Pol II to Dam-only binding peak at the same genomic location is indicated by red crosses. (B–D) Positive controls: Dam-Pol II occupancy of the genes expressed in the carpet glia. (B) moody locus, (C) spinster (spin) locus, and (D) pyramus (pyr) locus. Both biological replicates are shown in light and dark green. Scale bars on the y-axis represent the log2 ratio change between Dam-Pol II and Dam samples. [file Image_1.TIF]

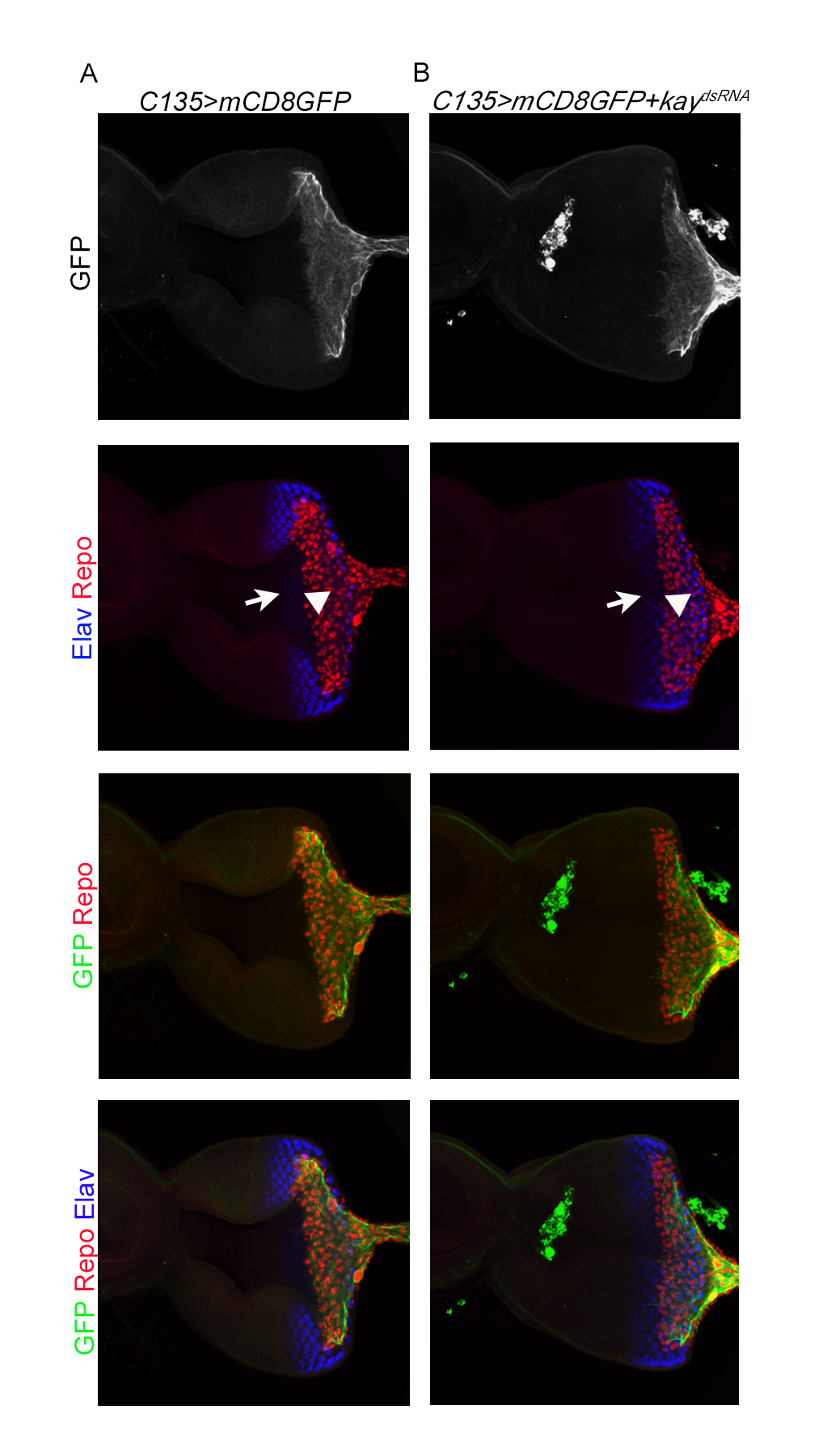

Supplement: Figure S2 — kay knockdown has no effect on carpet cell morphology. (A,B) All discs stained for the presence of carpet glia membrane (GFP staining, green), glial nuclei (Repo staining, red), and differentiated PRs (Elav staining, blue). The first row shows the membrane of carpet glia stained with GFP. The second row shows the relative position of the front of the PRs and that of the glia stained with Repo. White arrows and arrowheads in both A and B indicate Elav-stained PRs and Repo-stained glial cells, respectively. The third row shows the merged staining of Repo and GFP for the relative position between RBGs and the front of the carpet membrane, and the fourth row shows images merging all staining. [file Image_2.TIF]

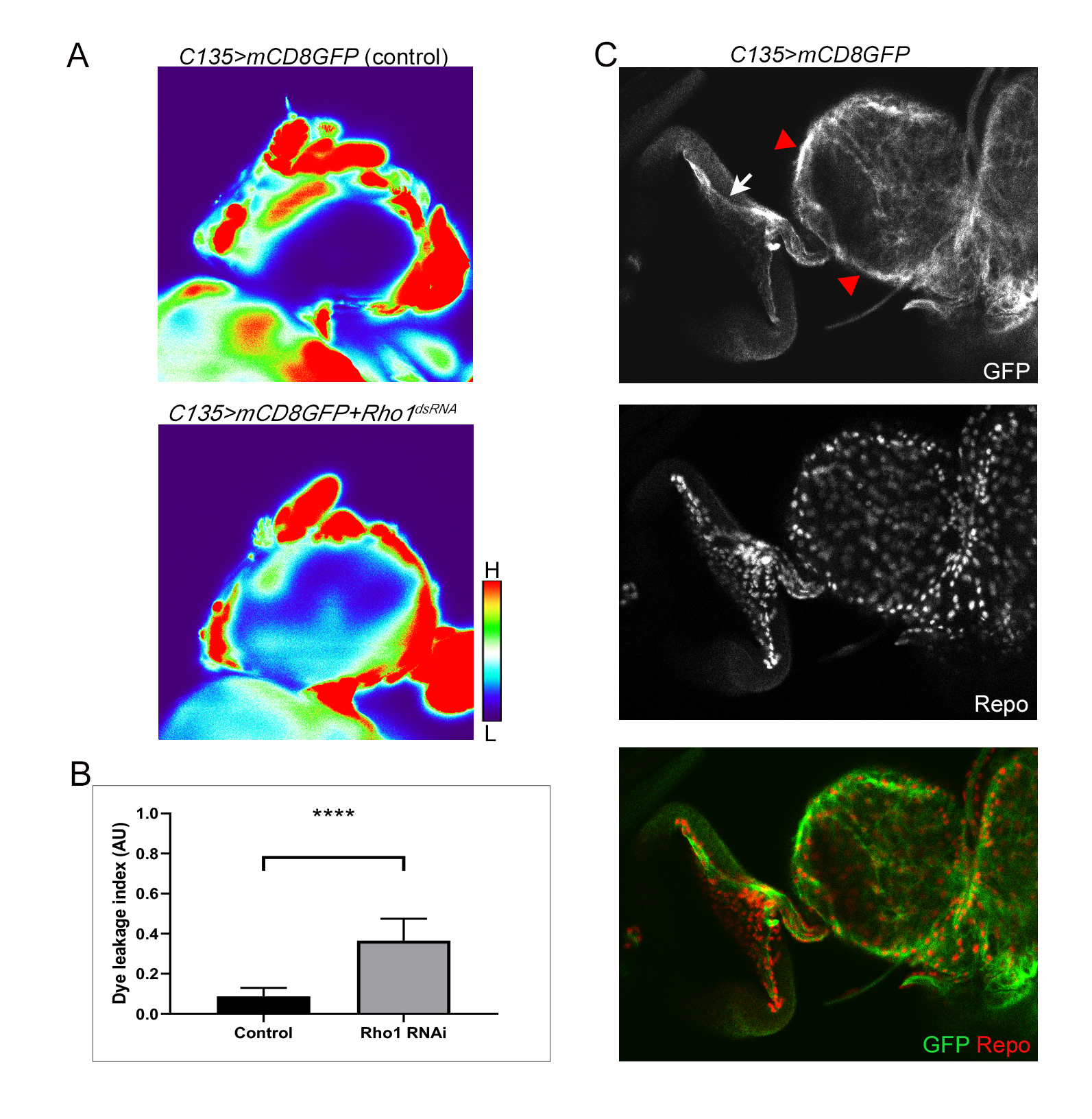

Supplement: Figure S3 — Blood–retina barrier permeability assay. (A) Confocal images of the heads of the control (upper) and Rho1 knockdown (lower) flies. (B) Quantification of the fluorescence intensity detected in each eye. Rho1 knockdown discs: n = 18; control discs: n = 11 (p < 0.0001, Student's t-test). (C) Expression of C135-Gal4 driving UAS-mCD8GFP in the larval brain and eye disc. The cell membrane of surface glia surrounding the larval brain, indicated by red arrowheads, and that of the carpet glia, indicated by white arrow, is marked by mCD8GFP (green). Glial nuclei are labeled by Repo (red). [file Image_3.TIF]
